# Enhancing Predictive Accuracy of Mood Symptoms Using Wearable Devices and Machine Learning in Bipolar Disorder

**DOI:** 10.2196/92172

**Published:** 2026-03-12

**Authors:** P Keerthana Nair, Priyanka Renita D'Souza

**Affiliations:** 1 Department of Psychiatry, Kasturba Medical College Mangalore, Manipal Academy of Higher Education, Manipal, India

**Keywords:** machine learning, wearable device, bipolar disorder, mood disorder, digital mental health

 We read the paper “Using Wearable Device and Machine Learning to Predict Mood Symptoms in Bipolar Disorder: Development and Usability Study” by Wu et al [1] with great interest. The integration of wearable data with machine learning to predict mood symptoms is clinically relevant and holds significant potential for real-world applications. However, we would like to highlight specific methodological aspects and possible future directions that could significantly enhance clinical reliability.

Our primary observation concerns the use of weekly mood assessments with backfilling. The authors acknowledged that their labeling method relied on self-rated questionnaires with backfilled mood symptoms for the preceding 7 days, and the participants may not complete these promptly as symptoms emerged, which could affect the accuracy of mood labels. In line with this, we appreciate the intent to minimize patient burden, but using retrospective entry to accurately capture daily mood fluctuations may not be reliable in the context of bipolar disorder. A patient feeling particularly low at the time of filling the weekly scale may unknowingly project that state onto the entire week, and that single net rating may not reflect the preceding days. This may create a risk of labeling daily physiological data with an inaccurate mood state, especially as the wearable data (sleep, activity, heart rate) are collected daily. To address this, future study designs may incorporate ecological momentary assessment, similar to the studies by Schwartz et al [2] and Li et al [3] that used digital biomarkers in studies on mood disorders. Implementing a quick visual check-in once or twice a day, for instance, a 5-point emoji scale with an optional short note, would reduce recall bias and ensure that daily wearable data are mapped against actual daily mood.

Furthermore, physiological markers such as average heart rate and total sleep hours are nonspecific and easily influenced by external stressors, including daily conflicts, work pressure, or life events. Without accounting for these confounding factors, the model risks interpreting situational stress as mood signals. As noted by the authors, heart rate is an index of sympathetic and parasympathetic influences of the autonomic nervous system in both stress and resting states, highlighting the overlap between stress response and mood states. Therefore, including a brief daily measure in future models to capture perceived daily stress would help in differentiating reactive distress from endogenous mood changes.

In conclusion, the integration of wearable biosensors is a promising development. However, we believe that a shift in study design from retrospective weekly aggregates to real-time, context-sensitive monitoring would further maximize the clinical utility of these models.

## References

[1] Wu CT, Hsieh MH, Chen IM (2025). Using wearable device and machine learning to predict mood symptoms in bipolar disorder: development and usability study. JMIR Med Inform.

[2] Schwartz S, Schultz S, Reider A, Saunders EFH (2016). Daily mood monitoring of symptoms using smartphones in bipolar disorder: a pilot study assessing the feasibility of ecological momentary assessment. J Affect Disord.

[3] Li H, Mukherjee D, Krishnamurthy VB (2019). Use of ecological momentary assessment to detect variability in mood, sleep and stress in bipolar disorder. BMC Res Notes.

